# A Case Report of Umbilical Vein Varix with Thrombosis: Prenatal Ultrasonographic Diagnosis and Management

**DOI:** 10.1155/2019/7154560

**Published:** 2019-08-06

**Authors:** Yuuki Matsumoto, Akihiro Yanai, Saori Kamei, Ayaka Yamaguchi, Hirokazu Nakamine, Kohei Fujita

**Affiliations:** ^1^Department of Obstetrics and Gynecology, The Japan Baptist Hospital, Kyoto, Japan; ^2^Department of Pathology, The Japan Baptist Hospital, Kyoto, Japan

## Abstract

Umbilical vein varix (UVV) is a very rare cord anomaly associated with intrauterine fetal death and fetal anomaly. We describe a case of extra-abdominal UVV with thrombosis. UVV was diagnosed at 23 weeks of gestation for the first time by ultrasonographic screening. Peak systolic velocity (PSV) near the UVV was partially increased up to about 100 cm/s, and blood flow was not detected in one of the umbilical arteries at 28 weeks of gestation. Therefore, the mother was hospitalized to monitor alterations of the PSV of the UVV frequently. Because the PSV of the UVV showed a sudden rapid increase up to about 150 cm/s at 32 weeks of gestation, she underwent emergent cesarean section on the same day to avoid sudden umbilical cord occlusion. The infant's birth weight was 1,744 g, and the Apgar scores were 8 and 9 at 1 and 5 minutes, respectively. The pathological examination showed UVV with thrombosis and an occlusion in one of the umbilical arteries. The neonatal laboratory data showed no coagulopathy. Based on our experience with this case, frequent ultrasonographic examination should be performed to detect the acute thrombosis in the case of extra-abdominal UVV, especially during the preterm period.

## 1. Introduction

Umbilical vein varix (UVV), variceal dilatation of the umbilical vein, is one of the rare umbilical anomalies associated with intrauterine fetal death and fetal malformations [1, 2]. Also, that can cause complications like thrombosis in the varix and neonatal coagulopathy [3, 4]. It indicates the blood flow inside UVV can change dynamically. Therefore, observation of change progress of UVV is important when you manage the patients.

Here, we describe a case of extra-abdominal UVV with thrombosis that might have caused occlusion of one of the umbilical arteries. Because of immaturity of the baby, we carefully observed conservatively 9 more weeks until changes occurred. She underwent emergency cesarean section and a male baby was born without any complications.

## 2. Case Presentation

**Figure 1:**
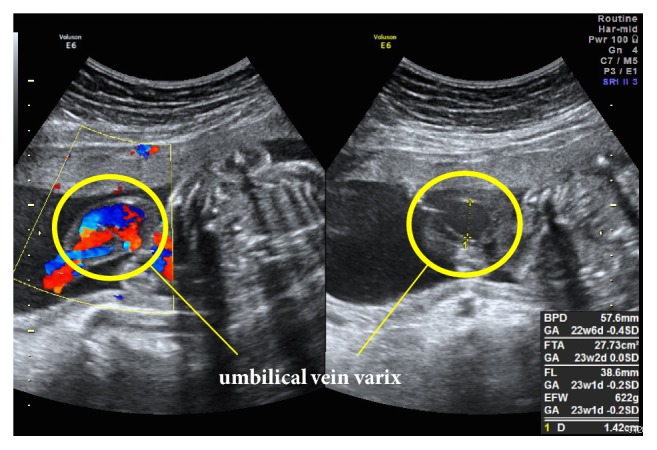
Ultrasonogram at 23 weeks of gestation shows abnormal bean-like dilation of the umbilical vein.

**Figure 2:**
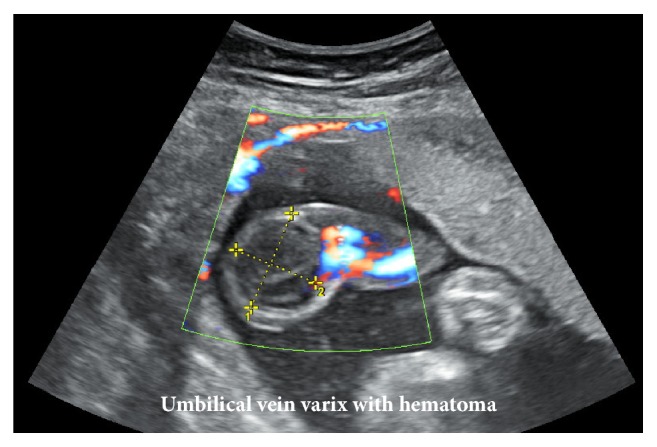
Ultrasonogram at 28 weeks of gestation shows enlargement of the thrombus or hematoma (measured 29 × 24 mm) beside the umbilical vein varix (UVV).

A 28-year-old woman (gravida 2, para 0) without relevant medical history became pregnant by artificial insemination with her husband's sperm and visited our hospital at 10 weeks of gestation. At 23 weeks of gestation, fetal screening ultrasonography showed two normal umbilical arteries and abnormal bean-like dilation of the umbilical vein (Figure 1), which was diagnosed as extra-abdominal UVV. Doppler ultrasonograms revealed bidirectional turbulent blood flow inside the varix. At 28 weeks of gestation, the hematoma beside the UVV enlarged up to 29 × 24 mm (Figure 2) and blood flow in one of the umbilical arteries was not detected, while peak systolic velocity (PSV) in another umbilical artery near the UVV increased up to about 100 cm/s. Because of fetal immaturity and the risk of umbilical blood flow interruption, we hospitalized her after obtaining informed consent and performed ultrasound screening once every two days to obtain a profile of the umbilical cord blood flow and ensure fetal well-being. The maternal blood-coagulation system was within the normal range.

**Figure 3:**
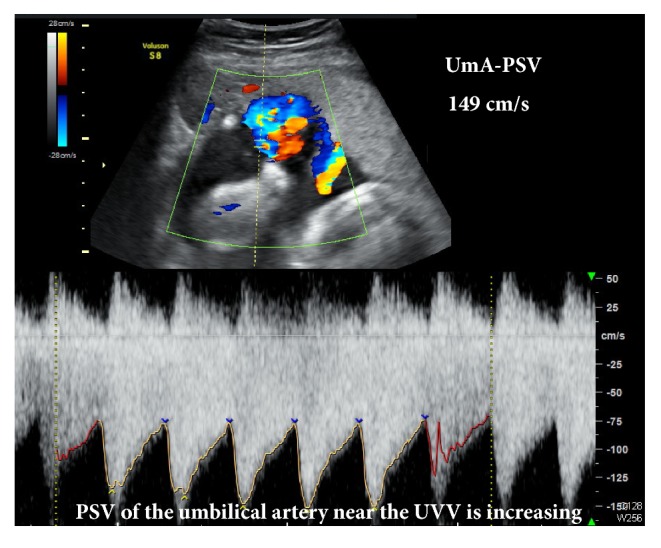
Ultrasonographic echocardiogram at 32 weeks of gestation shows edematous enlargement of the umbilical vein varix and an increase of the peak systolic velocity of the umbilical artery (UmA-PSV) up to 149 cm/s.

**Figure 4:**
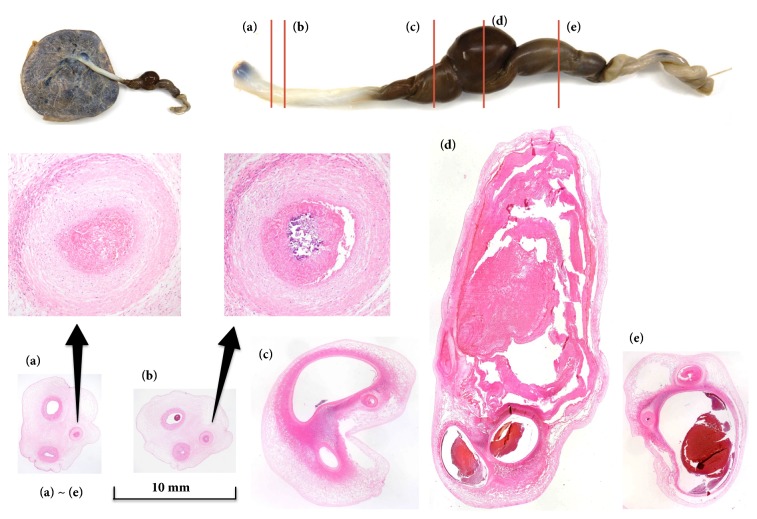
Pathological findings. In the middle of the umbilical cord, there is an area of vascular engorgement (125 mm in length) with central UVV. Cross-section of the cord shows occlusion of one of the umbilical arteries by a fibrin thrombus (a) with central calcification (b). Umbilical vein on both sides of the UVV is markedly dilated (c, e). The UVV, measured 25 x 11 mm, contains a large fresh thrombus (d).

At 32 weeks of gestation, edematous enlargement of the UVV and an increase of PSV of the UVV up to 149 cm/s were detected by ultrasonographic echocardiogram (Figure 3), and fetal heart rate tracing showed variable deceleration. No fetal abnormalities such as anemic changes (hydrops or an increase of the PSV of the middle cerebral artery) were suspected. On the same day, we performed emergent cesarean section to avoid additional acute risks of umbilical cord occlusion. The male baby was born with a birth weight of 1,744 g with Apgar scores of 8 (1 minute) and 9 (5 minutes), and umbilical arterial pH of 7.340. The pathological examination showed UVV with fresh thrombi, venous dilatation on both sides of the UVV, and occlusion of one of the umbilical arteries by fibrin thrombi with focal calcification (Figure 4). There was no abnormality in the placenta. The neonatal laboratory data showed no coagulopathy, and he had a good course after delivery.

## 3. Discussion

UVV or variceal dilatation of the umbilical vein is one of the umbilical anomalies that occur in either intra- or extra-abdominal portion of the umbilical cord [2]. Some case reports indicate UVV is associated with thrombosis in the varix, stillbirth, fetal anomaly, and neonatal coagulopathy [3, 4], though generally the outcome of most cases with isolated UVV is fair [5]. Extra-abdominal UVV is rarer than intra-abdominal UVV, and there have been only four reports regarding the diagnosis of extra-abdominal UVV with hematoma [2, 4, 6, 7]. In two cases, a diagnosis of UVV was made at 32 and 35 weeks of gestation [4, 7]. In the other two cases, the diagnoses were obtained after birth with one case being a stillbirth [6]. The babies in each case did not have any malformations.

In our case, frequent ultrasound examinations could reveal increasing acute massive thrombosis in the varix and that in one umbilical artery before delivery. The pathological examination showed massive nonorganized thrombi and hematoma in and around the UVV and occlusion of one of the umbilical arteries by fibrin thrombi with focal calcification. These findings were compatible with those of the prenatal ultrasonography, and they suggest that the UVV and hematoma around it laterally compressed the umbilical artery, resulting in its occlusion and thrombus formation. In this regard, frequent ultrasonographic examinations were useful in deciding whether to perform emergent cesarean section before occurring umbilical cord complications including umbilical venous embolism [8]. Our neonate followed a favorable course and did not have any abnormal findings such as coagulopathy that most likely occur in cases of such UVV thrombosis [4, 9].

It has been noted that fetal death tended to occur rapidly after an abnormal fetal nonstress test result [3]. Some studies have asserted that delivery is recommended as soon as the lungs of the fetus mature to reduce the risk of interrupting the umbilical cord blood flow [7, 10]. However, to date, there is controversy regarding how often the examination should be performed to determine the best term for termination of pregnancy before the onset of fetal distress or death by UVV with thrombosis, especially in the case of early preterm, as in this case. Weissmann-Brenner* et al.* recommends that serial ultrasonographic scans are required weekly from diagnosis to 28 weeks, and fetal cardiac monitoring along with the scans twice a week thereafter [5].

This is the first reported case of an infant with extra-abdominal UVV associated with thrombosis who could be monitored in detail by ultrasonographic examinations for such a long period of time (nine weeks) in the preterm stage. Based on our experience, we suggest that frequent ultrasonographic examinations are useful for detecting acute increasing thrombosis in the case of extra-abdominal UVV.
